# Differential Translation of *Dazap1* Transcripts during Spermatogenesis

**DOI:** 10.1371/journal.pone.0060873

**Published:** 2013-04-26

**Authors:** Chi-Kai Yang, Pauline Yen

**Affiliations:** 1 Graduate Institute of Life Sciences, National Defense Medical Center, Taipei, Taiwan; 2 Institute of Biomedical Sciences, Academia Sinica, Taipei, Taiwan; University of Nevada School of Medicine, United States of America

## Abstract

Deleted in AZoospermia Associated Protein 1 (DAZAP1) is a ubiquitous hnRNP protein that has been implicated in RNA transcription, splicing, and translation. It is highly expressed in testes, predominantly in late stage spermatocytes and post-meiotic spermatids. *Dazap1* deficiency in mice results in growth retardation and spermatogenic arrest. The gene produces two major transcripts of 2.4 and 1.8 kb, designated *Dazap1*-L and *Dazap1*-S, respectively. Results of our previous RNA *in situ* hybridization and immunostaining suggested translational regulation of the *Dazap1* transcripts during spermatogenesis. The main objectives of the study were to determine the origin of the two *Dazap1* transcripts and to investigate whether they were similarly translated. Our Northern and 3′ RACE analyses showed that the two transcripts were generated through alternative polyadenylation. In mouse testes, the levels of both transcripts were low at postnatal day 12 (P12), increased significantly at P18, and reached maximum at P27. Sucrose gradient analyses showed that at P12 both transcripts were actively translated. Afterward, an increasing portion of *Dazap1*-S became associated with the translationally inactive mRNPs, and the translational repression was accompanied by an increase in the length of its poly(A) tail. A much smaller portion of *Dazap1*-L was also sequestered to mRNPs as testes matured, but there was no changes in its poly(A) tail length. Using RNA pull-down followed by mass spectrometry, we identified DAZL, a germ-cell specific translation regulator, as one of the proteins that bound to the 3′UTR region specific for *Dazap1*-L. We further showed that DAZL preferentially bound to *Dazap1-*L in testis lysates and stimulated the translation of a reporter gene carrying *Dazap1*-L 3′UTR. In summary, our study shows that the translation of the two *Dazap1* transcripts is differentially regulated. It also provides a new example of translational repression associated with poly(A) tail elongation during spermatogenesis.

## Introduction

Most eukaryotic mRNAs are translated soon after they are processed and exported to the cytoplasm. However, some mRNAs are stored in the cytoplasm as inert messenger ribonucleoprotein particles (mRNPs) for a substantial period of time before they are translated. Such translation repression or mRNA masking is most frequently observed during gametogenesis and early embryogenesis [1]. A classic example of translational regulation during spermatogenesis is the *Prm1* gene (encoding protamine 1) which is transcribed in round spermatids and translated several days later in elongated spermatids [2]. Storage of *Prm1* mRNA for later translation is a necessary step for male germ cell development because protamine is needed at a stage when the nuclear transcription has been shut down. Premature translation of *Prm1* mRNA in round spermatids results in precocious condensation of nuclear DNA and dominant male sterility [3]. Translational repression of *Prm1* mRNA is mediated through its 3′UTR, and several proteins that bind to *Prm1* 3′UTR have been identified although how they regulate the translation remains unclear [4], [5], [6], [7]. Additional patterns of gene repression are also observed in spermatogenic cells [8]. Some mRNAs appear to be partially repressed at all time, while others are almost totally inactivated in free mRNPs and generate little proteins. Recent profiling of testicular mRNAs in polyribosomes (polysomes) and mRNP fractions of prepuberal and adult mice identified over seven hundreds of transcripts that are differentially translated during testis development [9].

Deleted in AZoospermia (DAZ) proteins are a family of RNA-binding proteins that regulate mRNA translation in germ cells [10]. They include DAZ, a spermatogenic factor encoded by the *AZFc* region of the Y chromosome that is frequently deleted in azoospermic men [11], and the autosomal encoded DAZL and BOULE [12], [13], [14]. DAZ proteins are expressed predominantly, if not exclusively, in germ cells and their deficiencies impair reproduction [15], [16]. A role of DAZ proteins in mRNA translation is supported by their presence on polysomes [17], [18] and their effects on translation in cultured cells [18], intact oocytes [19], [20] and zebrafish embryos [21]. A recent study using tethered function assay in *Xenopus* oocytes suggests that DAZL facilitates translation initiation by binding to the 3′UTR of its target mRNAs to recruit poly(A) binding proteins (PABPs) and stimulate the formation of 80 S ribosomes [19]. DAZL recognizes GUU triplet in different sequence context [18], [22], [23], and several putative natural substrates of DAZL have been identified though some of them need further verification *in vivo* [20], [24], [25], [26].

DAZ Associated Protein 1 (DAZAP1) is a DAZ interacting protein that is expressed ubiquitously but most abundantly in the testis [27], [28]. It is a component of the hnRNP particles [29] and has been implicated in several cellular processes including RNA transcription [30], [31], splicing [32], [33], [34], and translation [35]. Very recently, *DAZAP1* was suggested as a potential disease gene for amyotrophic lateral sclerosis [36]. Most mice deficient in DAZAP1 die perinatally, and the few survivors manifest growth retardation and spermatogenic arrest [37]. In mouse testes, DAZAP1 first appears abundantly in the nuclei of mid-pachytene spermatocytes, whereas our previous RNA *in situ* hybridization detected high levels of *Dazap1* transcripts in spermatogonia and early spermatocytes [38]. We thus set out to investigate possible delayed translation of *Dazap1* transcripts during spermatogenesis. Our results showed that two *Dazap1* transcripts were generated through alternative polyadenylation and their differential translation during spermatogenesis involved poly(A) tail length alteration and DAZL binding.

## Results

### Two *Dazap1* transcripts are generated through alternative polyadenylation

**Figure 1:**
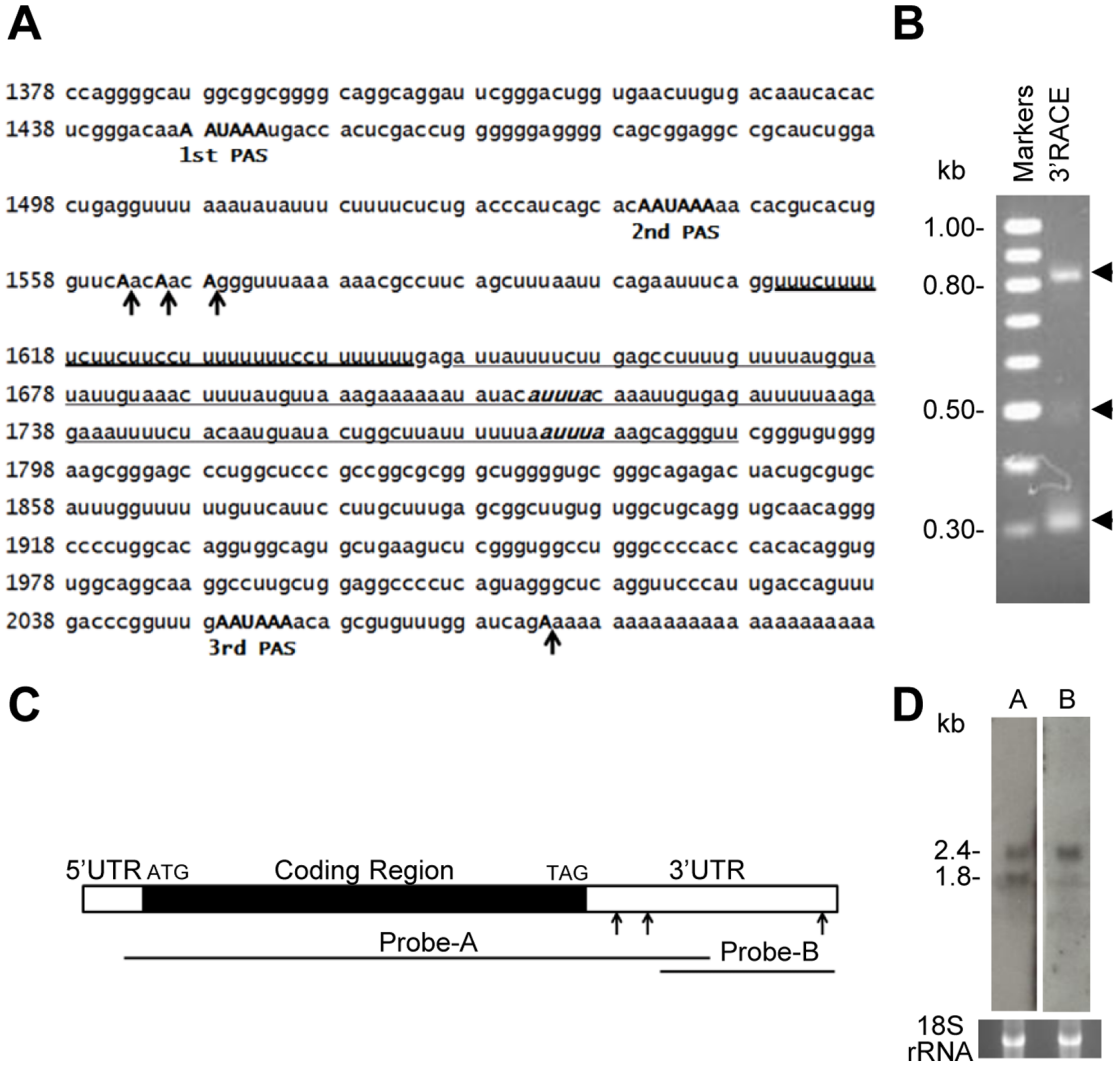
Alternative polyadenylation of the mouse ***Dazap1*** **transcripts.** (***A***) Sequence of the *Dazap1* 3′ UTR (NM_133188, nt1378–2093). The polyadenylation signals (PASs) are in bold capital letters, and the polyadenylation sites determined by 3′RACE are marked with arrows. The CU-rich region is indicated with heavy underline, and the AU-rich region with two AUUUA motifs (in bold italic) is indicated with light underline. (***B***) 3′RACE products of *Dazap1* transcripts in adult mouse testes. (***C***) Diagram of the *Dazap1* cDNA. The locations of Northern hybridization probes are indicated. The arrows point to the three PASs. (***D***) Northern analyses of *Dazap1* transcripts in adult mouse testes. Ethidium bromide (EtBr) staining of the 18 S rRNA is shown at bottom as a loading control.

Mouse testes express two major *Dazap1* transcripts of 2.4 and 1.8 kb, designated *Dazap1*-L and *Dazap1*-S, respectively [28]. We failed to detect alternative splicing and promoter usage by RT-PCR and 5′ Rapid Amplification of cDNA Ends (RACE), respectively, suggesting that the two *Dazap1* transcripts were generated through alternative polyadenylation (data not shown). The 3′UTR of *Dazap1* contains three canonical AATAAA polyadenylation signals (PASs) (Fig. 1A), but only the second and the third ones are evolutionally conserved throughout vertebrates (data not shown). To identify the polyadenylation sites of *Dazap1* transcripts, we performed 3′ RACE of adult mouse testis RNA and detected two major fragments around 0.8 and 0.3 kb (Fig. 1B). Sequencing of the fragments revealed that polyadenylation of the 0.3 kb fragment initiated from three CA sites downstream of the second PAS, whereas that of the 0.8 kb product started downstream of the third PAS (Fig. 1A). The minor 0.5 kb fragment represented a PCR artifact amplified from the 0.8 kb fragment since it was also produced by PCR amplification of a clone containing the 0.8 kb fragment. Thus the two *Dazap1* transcripts were produced through alternative polyadenylation utilizing the second and the third PASs. This was further confirmed by Northern blot (Fig. 1C, D). Probe-A which covers a large portion of the *Dazap1 *cDNA detected both transcripts, whereas probe-B which contains a segment downstream of the second PAS hybridized only to *Dazap1*-L.

### The *Dazap1* transcripts are differentially expressed during spermatogenesis

**Figure 2:**
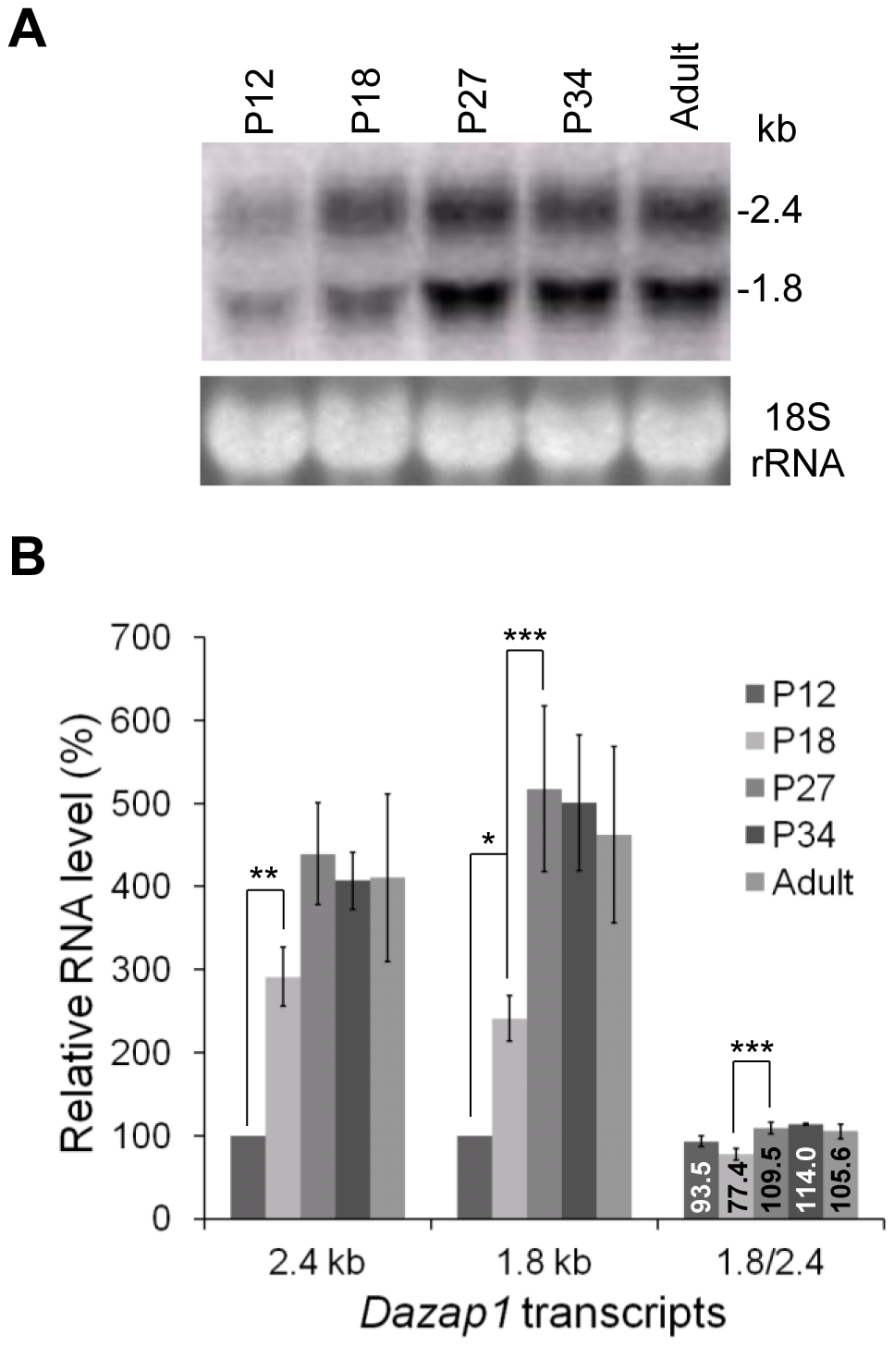
*Dazap1* expression during mouse testicular development. (***A***) Northern blot of total RNAs isolated from mouse testes at different postnatal ages. EtBr staining of the 18 S rRNA is shown as a loading control. (***B***) Quantification of the Northern blot signals. Radioactive bands on the hybridization members were excised and counted. Signals at P12 were used as the reference (100%). The results represent the averages of three independent experiments. Statistical significance of the differences was determined using one-way ANOVA. * denotes p<0.05, ** denotes p<0.01, *** denotes p<0.001. The relative levels (%) between 1.8 and 2.4 kb transcripts are given inside the bars.

We next studied the levels of the two *Dazap1* transcripts in prepuberal and adult mouse testes. Spermatogenesis is initiated soon after birth, and day 8 mouse testes contain only type A and type B spermatogonia [39]. Spermatocytes start to appear afterward. In postnatal day 12 (P12) testes, germ cells make up 60% of the total cell population and the most advanced germ cell is zygotene spermatocytes. At P18, pachytene spermatocytes dominate the germ cell population, and haploid spermatids start to appear after day 20. P27 testes are enriched in late round spermatids [40], and P34 and adult testes contain spermatids as the major cell types. Northern analyses of mouse testes at different ages showed that the levels of *Dazap1* transcripts were low at P12, increased significantly at P18, and reached the highest levels at P27 (Fig. 2A, B), consistent with *Dazap1* expression mainly in late pachytene spermatocytes and spermatids. The ratio of the two transcripts also changed as spermatogenesis progresses (Fig. 2B). The two transcripts were present in similar levels at P12 and P18, but *Dazap1*-S became the dominant form at P27 and after.

### The *Dazap1* transcripts are differentially translated in the testis

**Figure 3:**
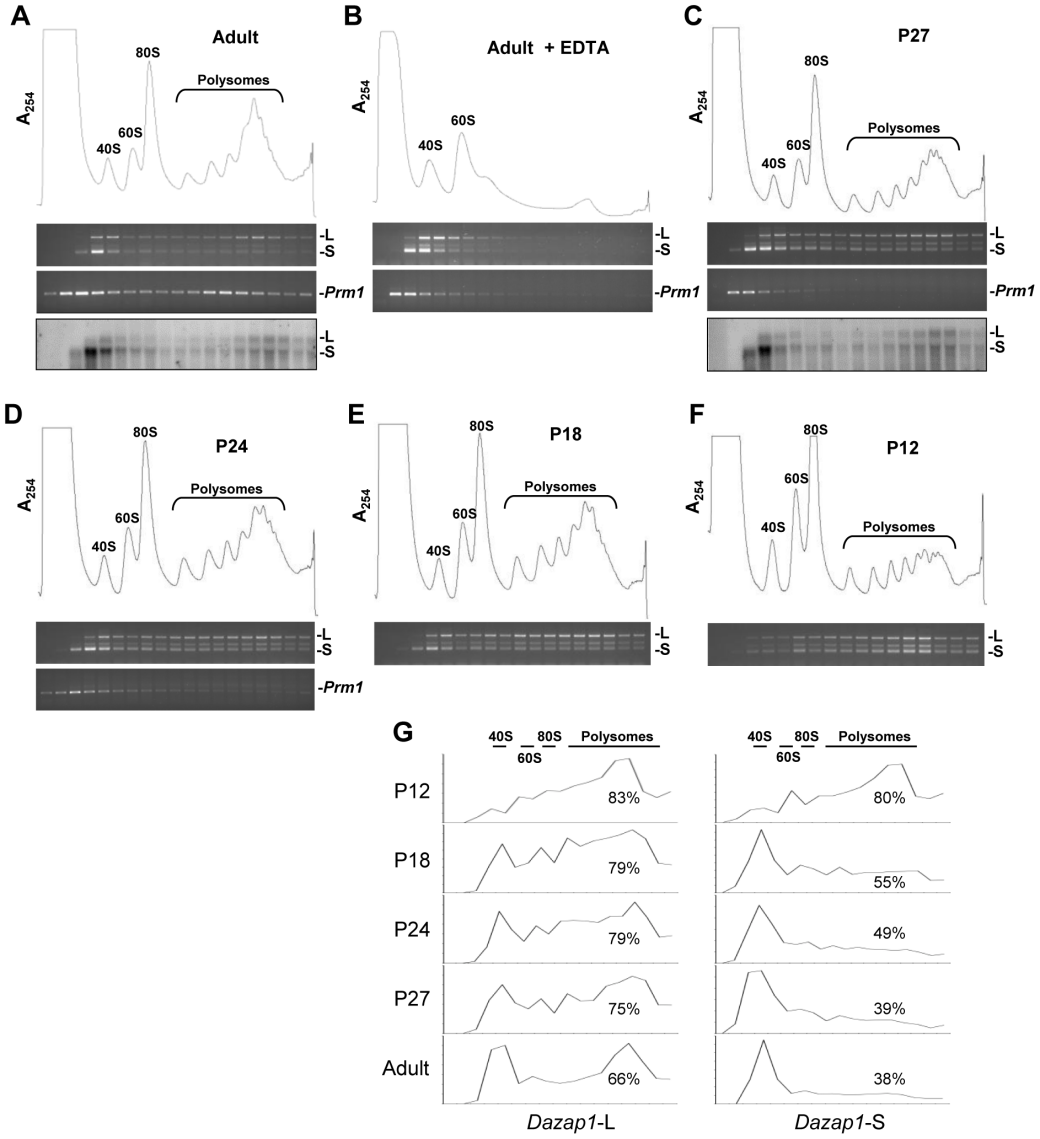
Sucrose gradient analyses of *Dazap1* transcripts in mouse testis lysates. Testis lysates were prepared from adult (***A*** **,** ***B***), P27 (***C***), P24 (***D***), P18 (***E***), and P12 (***F***) mice and fractionated on 10–50% sucrose gradients. The polysome profiles determined by optical absorbance at 254 nm (A_254_) are shown at top. *Dazap1*-L (L) and *Dazap1*-S (S) transcripts in the fractions were detected by 3′RACE (top gel panels) and Northern hybridization (bottom panels in ***A*** and ***C***), and the *Prm1* transcript was detected by RT-PCR. *Prm1* expression in P18 and P12 testes was undetectable. Quantification of the 3′RACE signals is shown in (***G***), with the percentage of total signals in the 80 S and polysomal fractions indicated.

To investigate whether both *Dazap1* transcripts are equally translated during spermatogenesis, we fractionated mouse testis lysates on 10–50% sucrose gradients and monitored the distribution of *Dazap1* transcripts by 3′RACE as well as Northern blot when *Dazap1* transcripts were sufficiently abundant for detection (Fig. 3). Both methods showed that in adult testes *Dazap1*-L had a bimodal distribution, with a large fraction associated with the polysomes, whereas a vast majority of *Dazap1*-S was present near the top of the gradient in the mRNP fraction (Figs. 3A, G). As a control, we also determined the distribution of the *Prm1* transcript and found it in both mRNP and polysomal fractions as expected [9]. Upon EDTA treatment, the polysomes dissociated and all *Dazap1* and *Prm1* transcripts were found near the top of the gradient (Fig. 3B). The polysomal profiles of *Dazap1* mRNAs in P27, P24, and P18 testes were similar to that of the adult testes, with a large fraction of *Dazap1*-S near the top of the gradients and most *Dazap1*-L associated with polysomes (Figs. 3C, D, E; G). On the other hand, the *Prm1* transcripts in P27 and P24 testes were present only in the mRNP fractions because the most advanced spermatogenic cells in these testes are round spermatids. At P18 and P12, *Prm1* transcripts were undetectable. There was a major change in the distribution of *Dazap1* transcripts in P12 testes, with both transcripts mainly associated with polysomes being actively translated (Figs. 3F, G). Since the major difference in germ cell constitution between P12 and P18 testes is the appearance of pachytene spermatocytes in the latter, our results suggest that after the meiotic germ cells reach the pachytene stage, a large fraction of *Dazap1*-S and a much smaller fraction of *Dazap1*-L start to be sequestered to mRNPs and become translationally inactive.

### Translationally repressed *Dazap1*-S has longer poly(A) tails

**Figure 4:**
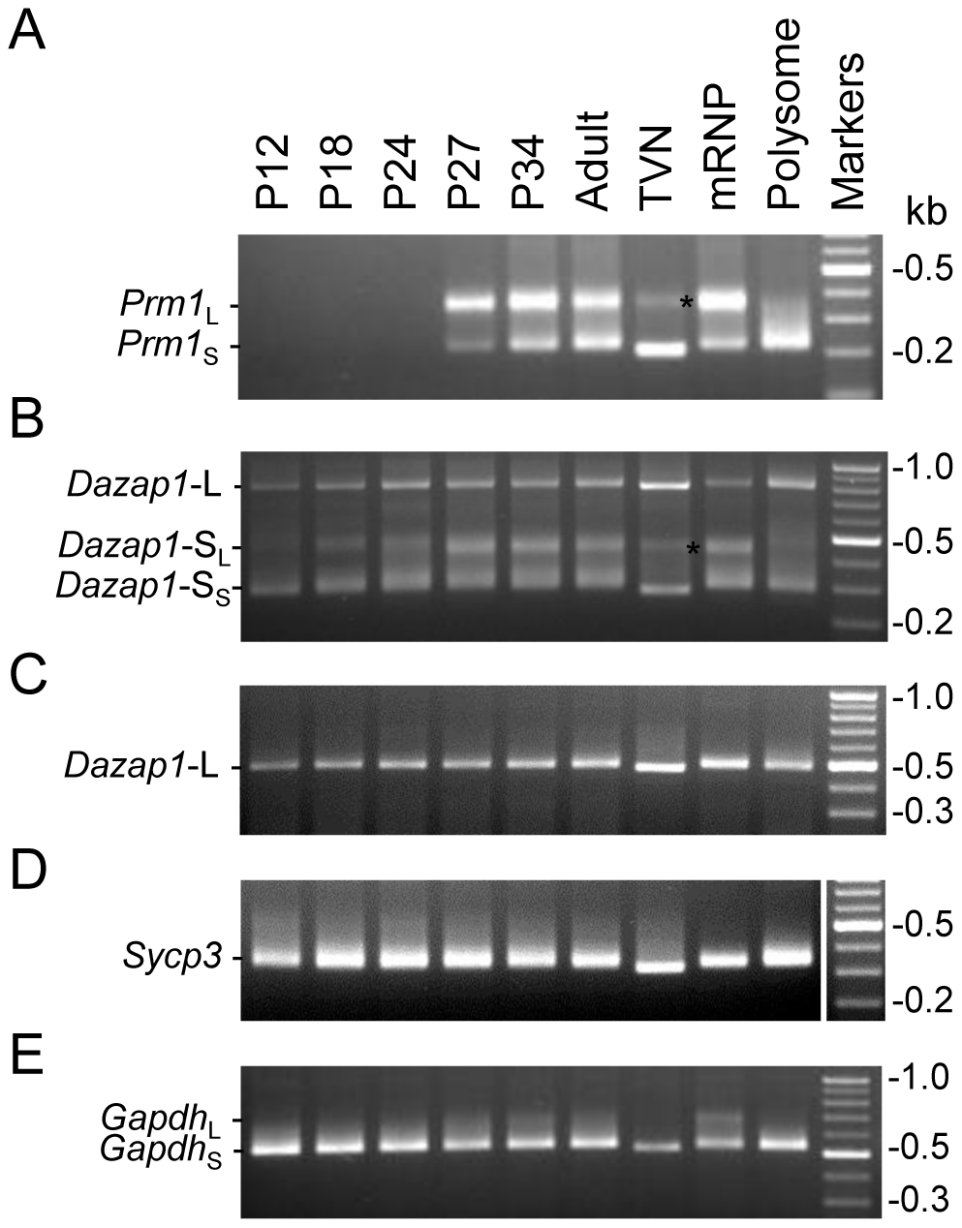
Developmental changes in the lengths of poly(A) tails of *Dazap1* and *Prm1* transcripts. Total testicular RNAs isolated from mice at the various ages and RNAs extracted from the free mRNP and polysomal fractions of sucrose gradients of adult testis lysates were subjected to ePAT analysis using forward primers specific for the various genes as indicated. In the TVN lanes, the major products contain an invariant 12-(A) poly(A) tail whereas the minor products, marked with asterisks, likely came from nonspecific amplification. Transcripts with long and short poly(A) tails are indicated with L and S subscript, respectively.

It has been reported that mouse *Prm1*, *Prm2*, *Tnp1*, and *Tnp2* transcripts being actively translated on polysomes have shorter Poly(A) tails than those that are translationally repressed [41]. To investigate whether translational regulation of *Dazap1* transcripts also involved changes in poly(A) tail length, we used a recently developed extension Poly(A) Test (ePAT) method [42] to monitor poly(A) tail lengths of *Dazap1* transcripts during mouse testis development and in sucrose-gradient fractions. Our initial test of ePAT on *Prm1* transcripts confirmed previous Northern hybridization results, i.e., translationally active polysomal *Prm1* transcripts had much shorter poly(A) tails than the major *Prm1* transcript present in the free mRNP fraction (Fig. 4A and Fig. S1). It is noticed that a small fraction of *Prm1* transcripts in the mRNPs contained short poly(A) tails. Based on the size of the TVN-PAT product that had an invariant poly(A) tail of 12 As as well as the sequences of five or more randomly selected ePAT clones (Table S1), the lengths of the poly(A) tails in the short and long *Prm1* transcripts were determined to be approximately 25 and 120 bases, respectively, compared with previous estimations of 30 and 150 bases [41]. We also found that when *Prm1* transcripts were first detected in P27 testes, the vast majority of them had long poly(A) tails (designated as LATs). As the testis matured to contain later stages of germ cells with active *Prm1* translation, the fraction of *Prm1* transcripts with short poly(A) tails (designated as SATs) increased progressively, consistent with translation activation of *Prm1* in elongated spermatids.

We next used a forward primer common to both *Dazap1* transcripts in the e-PAT reactions (Fig. 4B and Fig. S1). *Dazap1*-S also produced two major ePAT products with poly(A) tails approximately 30 and 170 bases long, respectively (Table S1). Before P27 most *Dazap1*-S had SATs. However, the level of *Dazap1*-S with LATs increased progressively as the mice grew older, and reached a level comparable to that of *Dazap1*-S with SATs at P27. Similar to the *Prm1* transcripts, *Dazap1*-S with both SATs and LATs were present in the translationally inactive mRNP fraction whereas only *Dazap1*-S with SATs was present in the polysomal fraction. In contrast, the poly(A) tail length of *Dazap1*-L remained unchanged during testis development. The tail was determined to be approximately 30 bases long using a different *Dazap1*-L specific forward primer that produced shorter ePAT fragments (Fig. 4C and Table S1). *Dazap1*-L in both mRNP and polysomal fractions had the same SATs. Additional ePAT reactions showed that the germ-cell specific *Sycp3* transcript also had constant SATs of approximately 30 bases (Fig. 4D and Table S1). On the other hand, the house-keeping *Gapdh* transcript produced a minor band representing LATs in the mRNP fraction, suggesting possible translation repression on a small fraction associated with poly(A) tail elongation (Fig. 4E). Our results therefore showed a correlation between poly(A) tail length and translational status for a subset of transcripts in the testis, with LATs of 120∼170 bases associated with translation repression.

### Several RNA-binding proteins bind to *Dazap1* 3′UTR

**Figure 5:**
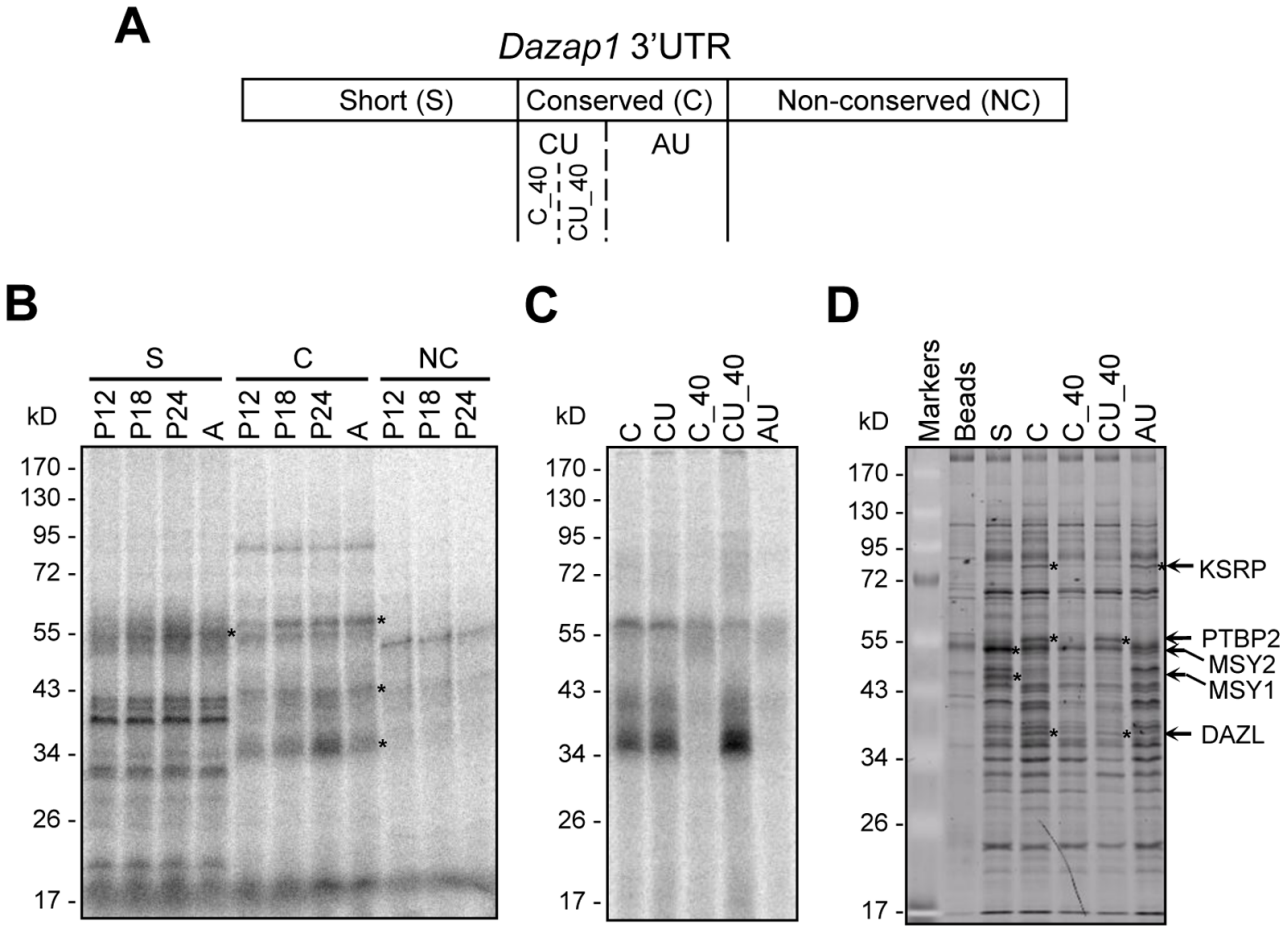
Identification of mouse testicular proteins binding to *Dazap1* 3′UTR. (***A***) Diagram of *Dazap1* 3′UTR showing the locations of the various RNA probes used in *B-D*. (***B***) UV cross-linking of ^32^P-labeled RNA probes from various regions of *Dazap1* 3′UTR and testicular proteins from mice at different ages. Asterisks point to proteins showing increasing expression during testis development. (***C***) UV cross-linking of probes from the C region and adult testis lysates. (***D***) RNA affinity pulldown assays of *Dazap1* 3′UTR fragments. Adult testis lysates were incubated with biotin-labeled RNAs and affinity purified by streptavidin-agarose beads. Bound samples were separated by 10% SDS-PAGE and proteins with specific binding were identified by LC/MS/MS spectrometry.

3′UTRs are known to contain *cis*-acting elements for the binding of translational regulators. We therefore searched for testicular proteins that bound to *Dazap1* 3′UTR. We separated *Dazap1* 3′UTR into three regions (Fig. 5A). The short (S) region (nt1378–1561) consists of the short 3′ UTR present in *Dazap1*-S; the conserved (C) (nt1568–1793) and the non-conserved (NC) (nt1788–2072) regions include the 5′ and 3′ segments, respectively, of the distal portion of *Dazap1* 3′UTR that is present only in *Dazap1*-L. RNA fragments corresponding to these regions were incubated with testis lysates prepared from mice at various ages, and the RNA-protein complexes were UV cross-linked and analyzed by SDS-PAGE (Fig. 5B). Probe S and probe C, but not probe NC, detected multiple proteins, and some of them showed increased binding during testis development.

We further separated the C region into the CU region (nt1568–1646) that contains a noticeable CU-rich sequence and the AU region (nt1644–1793) that contains several AU-rich elements (AREs), and dissected the CU region again into C_40 (not CU-rich, nt1568–1610) and CU_40 (nt1607–1646) subregions (Figs. 1A, 5A). UV cross-linking assays showed that most proteins detected by probe C bound to the CU_40 region (Fig. 5C).

We next performed RNA affinity pull-down assays followed by LC/MS/MS spectrometry to identify proteins that bound specifically to *Dazap1* 3′UTR. Using this approach we found several RNA-bind proteins, including MSY1 (Mouse Y box protein 1) and MSY2 (Mouse Y box protein 2) which bound to the S region, and KSRP (KH-type Splicing Regulatory Protein), PTBP2 (Polypyrimidine Tract-Binding Protein 2), and DAZL (Deleted in AZoospermia-Like) which bound to the C region (Fig. 5D). Additional pull-down experiments with smaller probes in the C-region showed that KSRP bound to the AU region whereas PTBP2 and DAZL bound to the CU_40 region.

### DAZL preferentially binds to *Dazap1*-L

**Figure 6:**
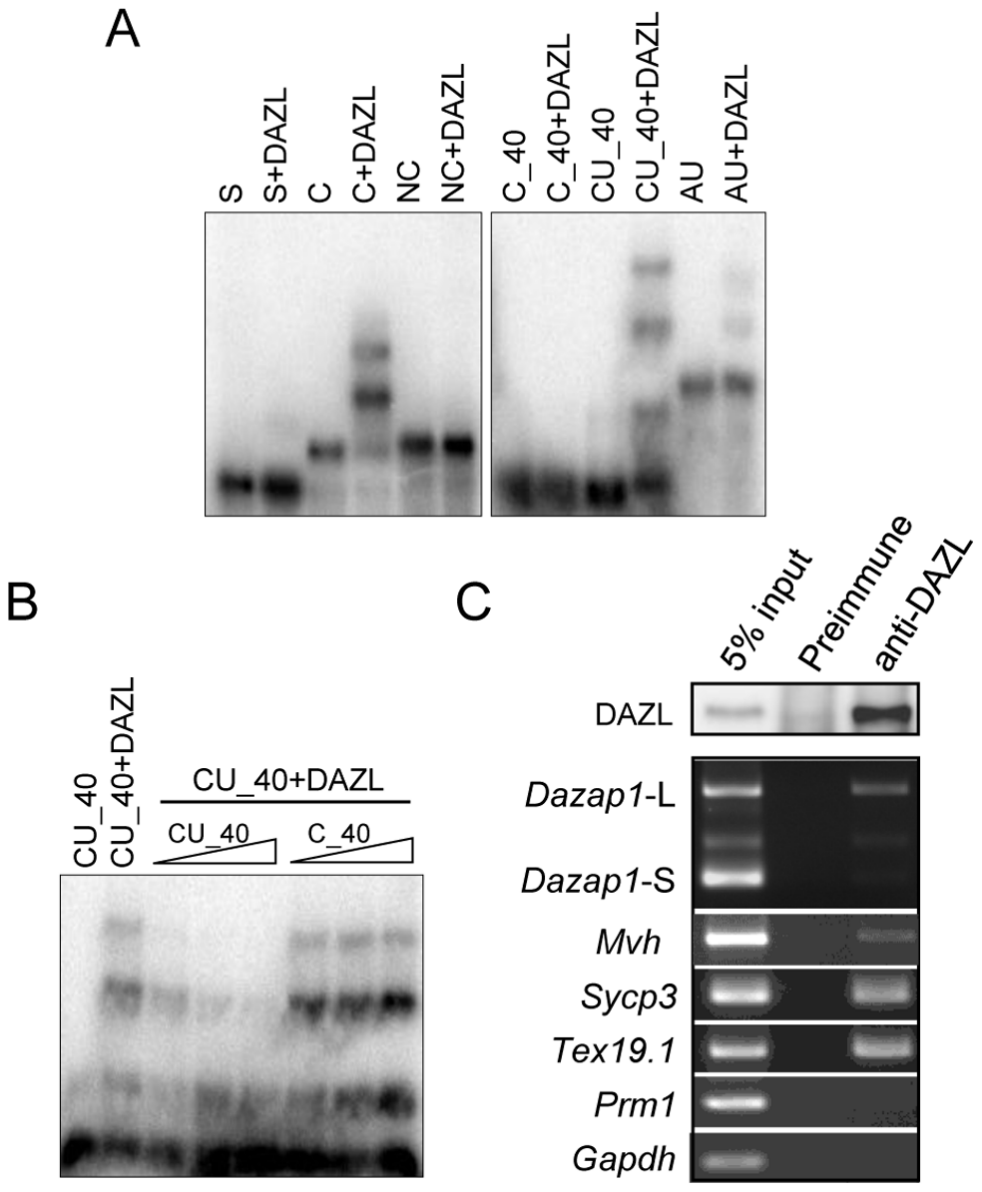
Specific binding of DAZL to the CU rich region in *Dazap1* 3′UTR. (***A***) RNA gel-shift assays. His-tagged DAZL was incubated with ^32^P-labeled RNA probes corresponding to various regions of *Dazap1* 3′UTR and analyzed on 1.5% agarose gels. DAZL binds to the CU_40 segment within the C region. (***B***) Competition of the binding between CU_40 and DAZL by increasing amounts (1, 5, 10 ng) of unlabeled RNA probes. (***C***) Preferential binding of DAZL to *Dazap1*-L in mouse testes. Adult mouse testes lysates were treated with a preimmune serum or an anti-DAZL antibody. The DAZL protein in the immunoprecipitates was detected by western blot with an anti-DAZL antibody (top panel). *Dazap1*-L and *Dazap1*-S in the precipitated RNAs were detected by 3′RACE, and the remaining transcripts were detected by RT-PCR with gene-specific primers (bottom panel).

DAZL has been reported to regulate the translation of several mRNAs in germ cells [10], and DAZL consensus binding sequences are present in different regions of *Dazap1* 3′UTR. We therefore performed RNA gel-shift assays using His-tagged recombinant DAZL to confirm the binding of DAZL to *Dazap1* 3′UTR (Fig. 6). We first mapped the DAZL binding site to the C region that is present only in *Dazap1*-L, and used smaller probes to narrow down the binding site to the CU_40 region which contains three partially overlapping copies of the DAZL consensus sequence (Fig. 6A). The binding between labeled CU_40 and DAZL could be competed out by increasing levels of cold CU_40 but not cold C_40, confirming the specificity of the binding (Fig. 6B).

### DAZL enhances the translation of a reporter with *Dazap1*–L 3′UTR

**Figure 7:**
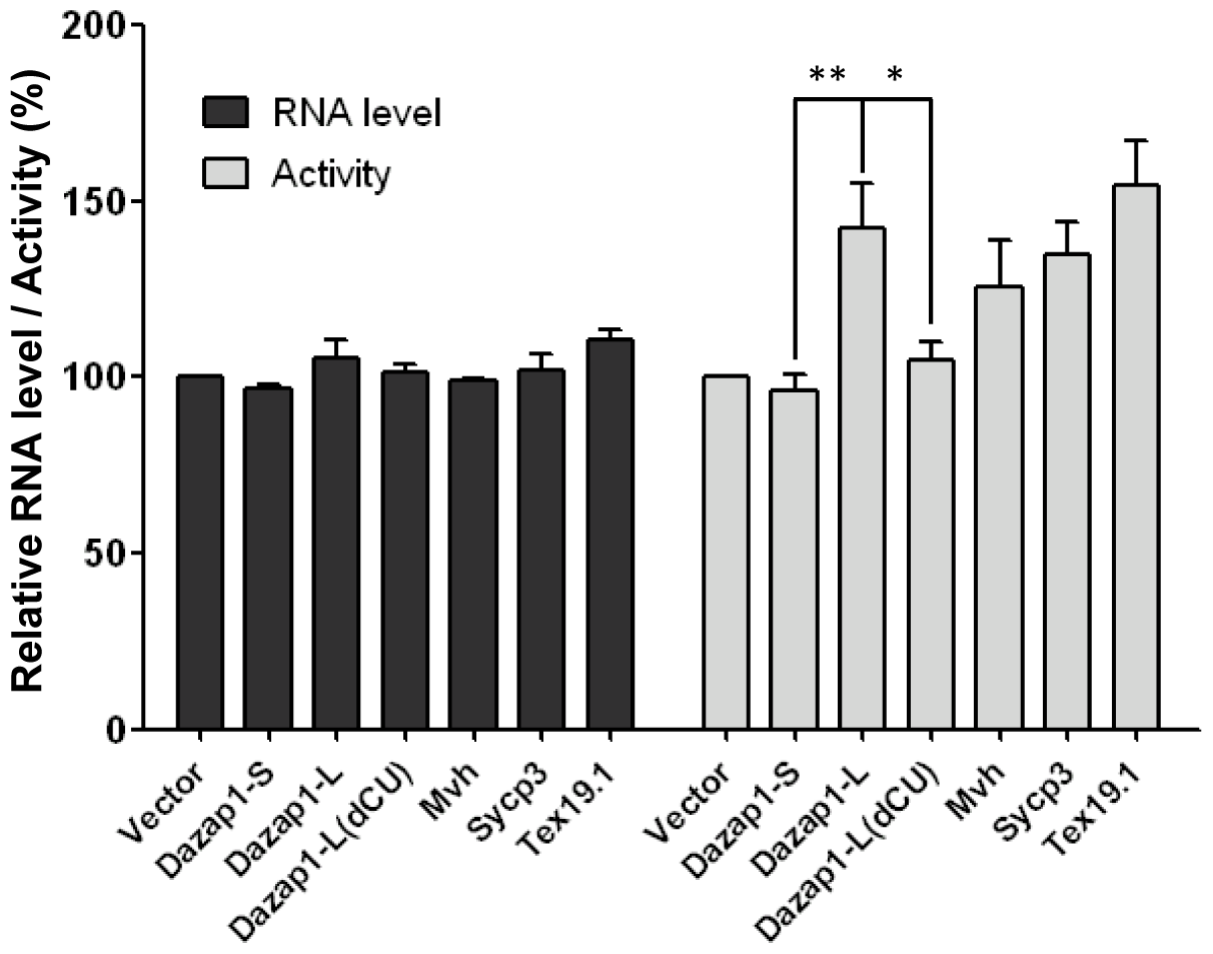
Enhancement of the translation of luciferase reporters by DAZL. 3T3 cells were transfected with a DAZL expression vector together with the empty *Luc* reporter vector (vector), or reporter vectors containing the 3′UTRs of the various transcripts. In *Dazap1*-L-dCU, the CU rich segment in the 3′UTR is deleted. The effects of DAZL on the luciferase activities in the transfected cells were determined. The *Luc* mRNA level and luciferase activity in cells transfected with the empty *Luc* vector were used as the reference (100%). The results shown represent the averages of three independent experiments. Statistical significance of the differences was determined using one-way ANOVA. * denotes p<0.05, ** denotes p<0.01.

We used luciferase reporter assays to indirectly study the effect of DAZL on the translation of *Dazap1* transcripts. We transfected 3T3 cells with a DAZL expression vector or the empty expression vector, together with the empty luciferase (*Luc*) reporter vector or a vector with *Luc* linked to the 3′UTRs of various genes. Western and RT-PCR analyses showed no significant differences in the levels of DAZL (Fig. S2) and *Luc* mRNA (Fig. 7) in the cells transfected with the DAZL expression vector and different *Luc* reporters. However, cells transfected with the reporter linked to the 3′UTR of *Dazap1*-L exhibited significantly higher luciferase activities than those transfected with the empty vector or the reporter linked to the 3′UTR of *Dazap1*-S (Fig. 7). Deletion of the entire CU-rich sequence from the *Dazap1*-L 3′UTR (*Dazap1*-L-dCU) abolished the translation enhancement. The degree of DAZL-induced translation enhancement of *Dazap1*-L was comparable to those of the reporters linked to the 3′UTRs of *Mvh*, *Sycp3*, and *Tex19.1*, which were much lower than what had previously been observed in other systems. DAZL was reported to increase approximately four-folds the translation of *Mvh* and *Sycp3* luciferase reporters in *Xenopu*s oocytes [25], [26] and about 10 folds that of a *Tex19.1* reporter in mouse oocytes [20]. Differences in the expression levels of DAZL and other trans-acting factors in the various systems may account for the disparity in the observed translational enhancement.

## Discussion

Alternative polyadenylation changes the content of the 3′UTR and offers a means to regulate gene expression at the post-transcriptional stage because most regulatory elements controlling the localization, stability and translation of a transcript are located in the 3′UTR [43]. Here we show that two *Dazap1* transcripts were generated through alternative polyadenylation, and that they were differentially expressed and translated during spermatogenesis. In P12 testes that contain spermatogonia and spermatocytes up to the zygotene stage, the transcripts were present at low levels and were both actively translated. However, with increased *Dazap1* expression at P18, there was a major shift in the polysomal profile of *Dazap1*-S, with an increasing portion of the transcript sequestered to translationally inactive mRNPs, whereas a large portion of *Dazap1*-L remained on polysomes. In P27 testes that contain mainly postmeiotic spermatids, the level of *Dazap1*-S increased significantly, consistent with previous EST analyses showing that round spermatids tend to have shorter truncated 3′UTRs [44]. Since most of the increased *Dazap1*-S was present in mRNPs, no increase in protein synthesis was expected and our previous immunostaining of mouse testicular sections also failed to detect significant increase in DAZAP1 signals in spermatids [38]. The situation is similar to that of the *Rnf4* transcripts encoding SNURF/RNF4 transcription modulator [45]. *Rnf4* has two transcripts of 3.0 and 1.6 kb that differ in their 3′UTRs. The 1.6 kb transcript is expressed abundantly in round spermatids but most of them are not associated with polysomes. It is unclear why germ cells spend their energy and resources to make mRNAs that are not used for protein production.

The Northern analyses on *Dazap1* expression in prepuberal testes, presented here, suggest that the expression of *Dazap1* expression in spermatogonia and early spermatocytes is much lower than that in pachytene spermatocytes. This contradicts the results of our previous RNA *in situ* hybridization on adult mouse testicular sections which showed highest levels of *Dazap1* mRNA in spermatogonia and early preleptotene spermatocytes [38]. We have since repeated the *in situ* experiments and were able to reproduce the same hybridization pattern with the same 5′ probe, but not with a probe from a different region of the *Dazap1* cDNA. We also purified different stage germ cells from adult mouse testes and found the highest levels of *Dazap1* transcripts in spermatocytes (data not shown). In addition, previous microarray analyses on purified spermatogenic cells also showed almost three times as much *Dazap1* transcripts in pachytene spermatocytes as in type A and type B spermatogonia [46]. Therefore our previous RNA *in situ* hybridization result likely represents an experimental artifact and there is no delayed translation of *Dazap1* transcripts as we previously suggested.

We investigated why *Dazap1*-S was translated on polysomes in early germ cells and sequestered to mRNPS in later germ cells, and found that translational repression of *Dazap1*-S was associated with an increase in its poly(A) tail length. Translational regulation mediated through poly(A) tail is well documented for some maternal mRNAs during oocyte maturation and early embryogenesis [47]. In the oocytes, masked mRNAs usually have short poly(A) tails fewer than 20 bases, which elongate to about 80 to 150 bases before the mRNAs are recruited to the polysomes for translation. It is thought that longer poly(A) tails facilitate the binding of poly(A) binding protein (PABP) and subsequent recruitment of translational initiation factors. Some translating mRNAs with long poly(A) tails, on the other hand, undergo deadenylation during oocytes maturation, resulting in their translational repression. As first reported by Kleene [41], male germ cells apparently have a reverse pattern of poly(A) tail mediated translational regulation, with the translational activation of several post-meiotically expressed genes accompanied by poly(A) shortening instead of lengthening. The shortened poly(A) tails still contain about 30 As, sufficient for one PABP molecule which requires approximately 27 As for binding [48], [49]. Here we confirmed poly(A) shortening of the *Prm1* transcript during translational activation using a recently developed ePAT method which offers higher sensitivity and resolution than the previously used Northern blot [41]. The *Prm1* short poly(A) tails gave a band that was quite discrete, suggesting that they may not be as heterogeneous as previously suggested. In addition, we found that a small fraction of the mRNP-bound *Prm1* transcripts contained SATs. Previous Northern analyses on sucrose gradient mRNAs enriched for transcripts with SATs did detect the presence of trace amount of *Prm1*, *Prm2*, *Tnp*1, and *Tnp2* transcripts with SATs in the mRNP fractions [50]. Our ePAT results are likely biased for transcripts with SATs since it is well known that in PCR reactions short fragments are usually amplified with higher efficiency. It remains unclear whether poly(A) shortening is required for translational activation or it is merely a consequence of translation [49], [51]. Our observations that a small fraction of the mRNP-bound *Prm1* transcript contained SATs whereas very little polysomal *Prm1* transcript contained LATs would argue for the occurrence of poly(A) tail shortening at mRNPs instead of on polysomes as a consequence of translation. The poly(A) tail elongation of *Dazap1*-S accompanying its translational repression represents the other side of the coin. Similar to the *Prm1* transcript, the mRNP-associated *Dazap1*-S had both LATs and SATs whereas the polysomal *Dazap1*-S contained only SATs. However for *Dazap1*-L which remained largely translationally active during spermatogenesis, there was no obvious changes in its poly(A) tail length. The transcript contained only SATs regardless whether it was sequestered to mRNPs or was actively translating on polysomes. Our results therefore showed that translational repression of *Dazap1*-S, but not *Dazap1*-L, was accompanied by poly(A) tail elongation. Whether the poly(A) tail plays an active role in modulating the translational activity of *Dazap1*-S remains to be investigated. Previous studies on testicular expression of *Ldh-x* and *Rpl32* failed to find an association between poly(A) tail length and translational activity [52], [53]. However, the Northern blots in those studies were not of high resolution/quality and the results need to be confirmed with the new ePAT method.

We have identified several proteins, including KSRP, PTBP2, MSY2, MSY1, and DAZL, that bound to *Dazap1* 3′UTR. The only protein that we found to have any effects on *Dazap1* expression when ectopically expressed in cultured cells was DAZL. DAZL is known to stimulate mRNA translation, and requires multiple DAZL binding sites for efficient translation activation [19]. DAZL consensus sequence U_2–10_[G/C]U_2–10_ is present in different regions of *Dazap1* 3′UTR. The S region contains a UUUCUUUU sequence, the CU_40 region contains three partially overlapping UUCUU sequences, and the AU region contains a UUUUCUU and a UUUUGUUUU sequence. Our RNA gel-shift assays showed that DAZL bound much stronger to the CU_40 region than the S or the AU region. Expression of luciferase reporters in 3T3 cells also indicated that the CU_40 region was responsible for the DAZL induced translation enhancement of the *Luc* gene linked to *Dazap1*-L 3′UTR. We also found that an anti-DAZL antibody preferentially precipitated *Dazap1*-L from mouse testis lysates. There is a good correlation between the expression of DAZL and DAZAP1 during spermatogenesis. Previous immunostaining of adult mouse testicular sections detected dramatic increases in the expression of both DAZAP1 and DAZL in pachytene spermatocytes [38], [54]. Our current analyses of *Dazap1* expression during spermatogenesis showed that the level of *Dazap1*-L also increased significantly in pachytene spermatocytes. Therefore, increased expression of both *Dazap1*-L and DAZL in pachytene spermatocytes likely accounts for the robust translation of DAZAP1 in the cells.

## Materials and Methods

### Animals

C57BL/6 mice used in the study were housed in a specific pathogen-free animal facility. Adult mice were 2–4 months old. The experiments were approved by Academia Sinica's Institutional Animal Care & Utilization Committee.

### 3′Rapid Amplification of cDNA Ends (3′RACE)

3′RACE of mouse *Dazap1* transcripts was carried out according Schaefer [55]. Testis RNA was reverse transcribed using the universal adapter primer PrRACE-dT: 5′-gctgtcaacgatacgctacgtaacgt_24_, and PCR amplified using primers PrDAP176: 5′-agtggcttcggacgcgggcagaaccacaac and Pr3′RACE: 5′-gctgtcaacgatacgctacgtaacg. The PCR products were cloned into pCRII-TOPO (Invitrogen, Carlsbad, CA) and sequenced.

### Northern hybridization

Northern hybridization was performed according to the standard protocol [56] using Sure Blot nylon membranes (Intergen, Purchase, NY) and a Stratalinker UV cross-linker 1800 (Stratagene, La Jolla, CA). Blots were hybridized with radioactive probes at 68°C in the ExpressHyb solution (Clontech, Palo Alto, CA) and analyzed with the Typhoon 9410 Imager (GE Healthcare, Piscataway, NJ). For quantification, the hybridization membranes were exposed to X-ray films and radioactive bands were located, excised, and counted.

### Sucrose gradient analysis

Post-mitochondrial fractions of mouse testis lysates were fractionated on 10–50% sucrose gradients as previously described [57]. Briefly, mouse testes were homogenized with a motor-driven glass–Teflon homogenizer (Wheaton, Millville, NJ) on ice in 0.5 ml lysis buffer (100 mM NaCl, 3 mM MgCl_2_, 20 mM Hepes, pH 7.4 and 0.5% Triton X-100). The nuclei and mitochondria were pelleted by centrifugation for 5 min at 12,000 *g*. The supernatants were loaded onto linear gradients of 10%–50% sucrose (w/w) in the lysis buffer without Triton X-100 and centrifuged for 2 h at 36,000 rpm in a SW-41 rotor (Beckman, Palo Alto, CA). The gradients were pumped through a flow cell (Teledyne Isco, Lincoln, NE) to record the absorbance at 254 nm and fractionated into 0.5-ml fractions. Each fraction was extracted twice with phenol:chloroform (1∶1) and the RNA was ethanol precipitated. Polysome profiles of the *Dazap1* transcripts were analyzed by 3′RACE-PCR or Northern hybridization. Control gradients, containing 16 mM EDTA in the postmitochondrial supernatants, were centrifuged and analyzed in parallel with the polysome gradients.

### UV cross-linking assays

Testis extracts were prepared from mice at indicated age according to Xu and Hecht [58]. ^32^P-labelled single stranded RNA probes were *in vitro* transcribed using Riboprobe systems (Promega) and purified on 4.5% denaturing polyacrylamide gels containing 8 M urea. UV cross-linking of RNA-protein complexes was performed according to Xu and Hecht [58] with minor modifications. RNA-protein binding was carried out at room temperature for 20 min in 20- µl reaction mixtures containing 20 mM Hepes [pH 7.6], 3 mM MgCl_2_, 40 mM KCl, 2 mM DTT, 5% [w/v] glycerol, 40 µg testis extracts, and 5×10^4^ cpm of ^32^P-labelled RNA probes. Heparin was then added to a final concentration of 5 mg/ml to reduce nonspecific RNA-protein interactions. After10 min, the reaction mixtures were exposed to UV light (Stratalinker 1800) for 11 min on ice, followed by RNase A (0.5 mg/ml) digestion for 15 min at 37°C. The RNA-protein complexes were resolved by 10% SDS-PAGE. The gels were dried and visualized with the Typhoon 9410 Imager.

### RNA affinity pulldown assays

RNA affinity pulldown assay was done according to a published protocol [58] with minor modifications. Biotinylated RNA probes were *in vitro* transcribed using the Riboprobe system containing biotin-11-UTP (PerkinElmer, Boston, MA). Testes extracts containing 1 mg total proteins were mixed with 20 µg biotinylated RNA in 400 µl binding buffer (BB) containing 20 mM Tris-HCl [pH 7.4], 2.5 mM MgCl_2_, 120 mM KCl, 1 mM DTT, 5% [w/v] glycerol, and1× protease inhibitor cocktail for 30 min at room temperature and treated with 5 mg/ml heparin for 15 min. Streptavidin-agarose beads were then added to the mixture and incubated for 60 min. After five washes with BB, bound proteins were released by boiling in 4× SDS sample buffer, resolved by 10% SDS-PAGE and visualized by SYPRO Ruby staining. The protein bands of interest were excised and analyzed by LC/MS/MS with a Thermo Scientific LTQ XL mass spectrometer (Thermo Scientific, Waltham, MA).

### RNA immunoprecipitation assays

RNA immunoprecipitation assay was performed according to Xu and Hecht [58]. Two mg testes extracts were precleaned using 50 µl protein G beads (GE Healthcare) and 100 µg/ml yeast tRNA in 1 ml TKM buffer (20 mM Tris [pH 7.4], 120 mM KCl, 1.5 mM MgCl_2_, 0.1% NP-40) with 500 U/ml SUPERase-In (Ambion, Austin,TX) for 4 hours at 4°C. After spinning down the beads, the supernatants were incubated overnight at 4°C with 10 µg anti-DAZL-C antibody which was generated against an oligopeptide containing the last 21 amino acid residues of mouse DAZL and affinity purified using the services of Bethyl Laboratories, Inc (Montgomery, TX). The mixtures were then incubated with 20 µl protein G beads for 3 hours at 4°C. The beads were spundown and washed five times by the TKM buffer. Immunoprecipitated RNAs were extracted by Trizol (Invitrogen) and analyzed by RT-PCR. Sequences of the PCR primers used in RNA immunoprecipitation assays are given in Table S2.

### RNA gel shift assays

Gel shift assays were carried out according to Venables et al [22] with some modifications. The 10- µl binding reactions contained 1 µg recombinant DAZL, 20 mM Tris [pH 7.4], 2.5 mM MgCl_2_, 60 mM KCl, 1 mM DTT, 5% [w/v] glycerol, and 10^4^ cpm of ^32^P-labelled RNA probes. After incubation at room temperature for 5 min, the reactions were treated with 5 mg/ml heparin and 0.5 mg/ml yeast tRNA for another 15 min. The RNA-protein complexes were separated on 1.5% agarose gels in 0.5× TBE buffer and visualized with the Typhoon9410 Imager.

### Plasmid construction

The entire 3′UTRs of *Dazap1, Mvh, Sycp3 and Tex19.1* were PCR amplified from mouse genomic DNA and cloned into pCRII-TOPO. pcDNA-Luc was generated by inserting the *Luc* gene into pcDNA3.1 (Invitrogen). The 3′UTRs of the various genes in pCRII-TOPO were then cloned into pcDNA-Luc to generate the reporter vectors. Deletion of the CU-rich sequence in *Dazap1* 3′UTR was generated by a PCR-mediated deletion approach [59]. For *in vitro* transcription, different regions of *Dazap1* 3′UTR were generated by PCR amplification and cloned into pBluescript (Stratagene). *Dazl* coding region was RT-PCR amplified from mouse testis RNA and cloned into pCRII-TOPO for sequencing. It was subsequently cloned into p3xFLAG-CMV-14 (Sigma, Saint Louis, MI) to generate pDAZL-Flag, and into pET-28a (Novagen, Madison, WI) to generate pET-DAZL for the production of His-tagged DAZL protein in *E. coli* [22], [60].

### Analysis of the translation of luciferase reporters

3T3 cells were maintained in DMEM (Hyclone, Fremont, CA) supplemented with 10% fetal bovine serum and 1× penicillin/streptomycin (GIBCO, Eggenstein, Germany). Transfection was performed using the TurboFect reagent (Fermentas, Burlington, Ontario, Canada). The pDAZL-Flag or the empty p3× FLAG-CMV-14 were co-transfected into 3T3 Cells with different firefly luciferase reporter constructs and pRL-TK (Promega, San Luis Obispo, CA) which encodes the *Renilla* luciferase. The cells were harvested after 24 hours, and luciferase activities in the cell lysates were measured using the dual-luciferase reporter assay system (Promega) and normalized to *Renilla* luciferase activities. Luciferase RNA levels were determined by semi-quantitative RT-PCR and normalized to the levels of *Renilla* luciferase transcripts. Luciferase activities and RNA levels in cells transfected with pDAZL-Flag were normalized to those in cells transfected with p3× FLAG-CMV-14 to reflect the effects of DAZL, and the results of cells transfected with different *Luc* reporter genes were compared using those of cells transfected with the empty *Luc* reporter as a reference (100%). DAZL expression levels were determined by Western blot and normalized to the levels of tubulin.

### Poly(A) tail length determination

A newly developed extension poly(A) test (ePAT) method was used to measure the poly(A) tail of mRNA [42]. This method makes use of the ability of the Klenow polymerase to extend the 3′ ends of RNA molecules with dNTPs from an annealed DNA template in standard reverse transcription buffers. The PAT-anchor primer 5′-cgctacgtaacggcatgacagtgt_22_ contains a unique universal DNA segment plus 22 Ts at the 3′ end and anneals to the end of poly(A) tails to serve as the DNA template for the Klenow polymerase. After the initial extension, the PAT-anchor primer also serves as the primer for the standard reverse transcription. The incubation was carried out at 55°C to eliminate unwanted priming from internal poly(A) tracts. The protocol started with heating 1 µg RNA together with 1 µl of 100 µM PAT-anchor primer in a volume of 8 µl at 80°C for 5 min followed by cooling down to 37°C. A master mixture containing 4 µl DEPC-treated water, 4 µl 5× Superscript III buffer (Invitrogen), 1 µl 100 mM DTT, 1 µl 10 mM dNTPs, 1 µl RNaseOUT (Invitrogen), and 1 µl (5 U) Klenow polymerase (New England Biolabs, Ipswich, MA) was added and the solution was incubated at 37°C for 1 hour. The reaction mixture was then heated at 80°C for 10 min to inactivate the polymerase and cooled down to 55°C. One µl (200 U) Superscript III (Invitrogen) was added and incubation was kept at 55°C for 1 hour. The reverse transcriptase was inactivated at 80°C for 10 min at the end of the reaction. PCR amplification of the 3′ ends of specific genes was performed using a universal reverse primer 5′-cgctacgtaacggcatgacagtg complementary to the 5′ end of the PAT-anchor primer and gene-specific forward primers, the sequences of which are provided in Table S3. A separate TVN-PAT reaction was also performed to generate a fragment with a defined poly(A) tail of 12 As. It served as a size standard for other PAT products that were amplified from the same gene specific forward primer. The TVN-PAT primer has an identical sequence as the PAT anchor primer except the addition of two 3′ variable bases V and N (V is A, G or C and N is any base) which fix the primer to the polyadenylation site during reverse transcription. The e-PAT products were analyzed on 2% agarose gels. Fragments of interest were purified from the gels, cloned into pCRII-TOPO and sequenced to determine the lengths of the poly(A) tails.

## Supporting Information

Figure S1 **Poly(A) tail length determination of** ***Prm1*** **and** ***Dazap1*** **transcripts in sucrose gradient fractions.** Adult mouse testis lysate was fractionated on a 10–50% sucrose gradient and the same proportions of RNAs isolated from alternating fractions were subjected to ePAT and analyzed on a 2% agarose gel. The type of ribosome present in each fraction is indicated at the top. The major bands in the TVN lanes contain an invariant 12-(A) poly(A) tail. Transcripts with long and short poly(A) tails are indicated with L and S subscript, respectively.
(DOCX)

Figure S2 **DAZL expression level in luciferase reporter assays.** The upper panels show western blots of lysates of 3T3 cells cotransfected with pDAZL-Flag and different 3′UTR luciferase reporters. DAZL-Flag was detected with an anti-Flag antibody. The α-tubulin level is used as a loading control. The bottom panel shows quantification of the western blot signals. DAZL-Flag signals were normalized to the tubulin signals. The results represent the averages of three independent experiments.
(DOCX)

Table S1 **Poly(A) tail lengths of ePAT clones.**
(DOCX)

Table S2 **PCR primers for RNAs precipitated from mouse testis lysate with an anti-DAZL antibody.**
(DOCX)

Table S3 **Gene-specific ePAT forward primers.**
(DOCX)
