# Supplementary material for: Differential Translation of Dazap1 Transcripts during Spermatogenesis
Source: PLoS One. 2013 Apr 26;8(4):e60873. doi: 10.1371/journal.pone.0060873 (PMC3637229; doi:10.1371/journal.pone.0060873)
Supplement: Figure S1 — Poly(A) tail length determination of Prm1 and Dazap1 transcripts in sucrose gradient fractions. Adult mouse testis lysate was fractionated on a 10–50% sucrose gradient and the same proportions of RNAs isolated from alternating fractions were subjected to ePAT and analyzed on a 2% agarose gel. The type of ribosome present in each fraction is indicated at the top. The major bands in the TVN lanes contain an invariant 12-(A) poly(A) tail. Transcripts with long and short poly(A) tails are indicated with L and S subscript, respectively. (DOCX) [file pone.0060873.s001.docx]

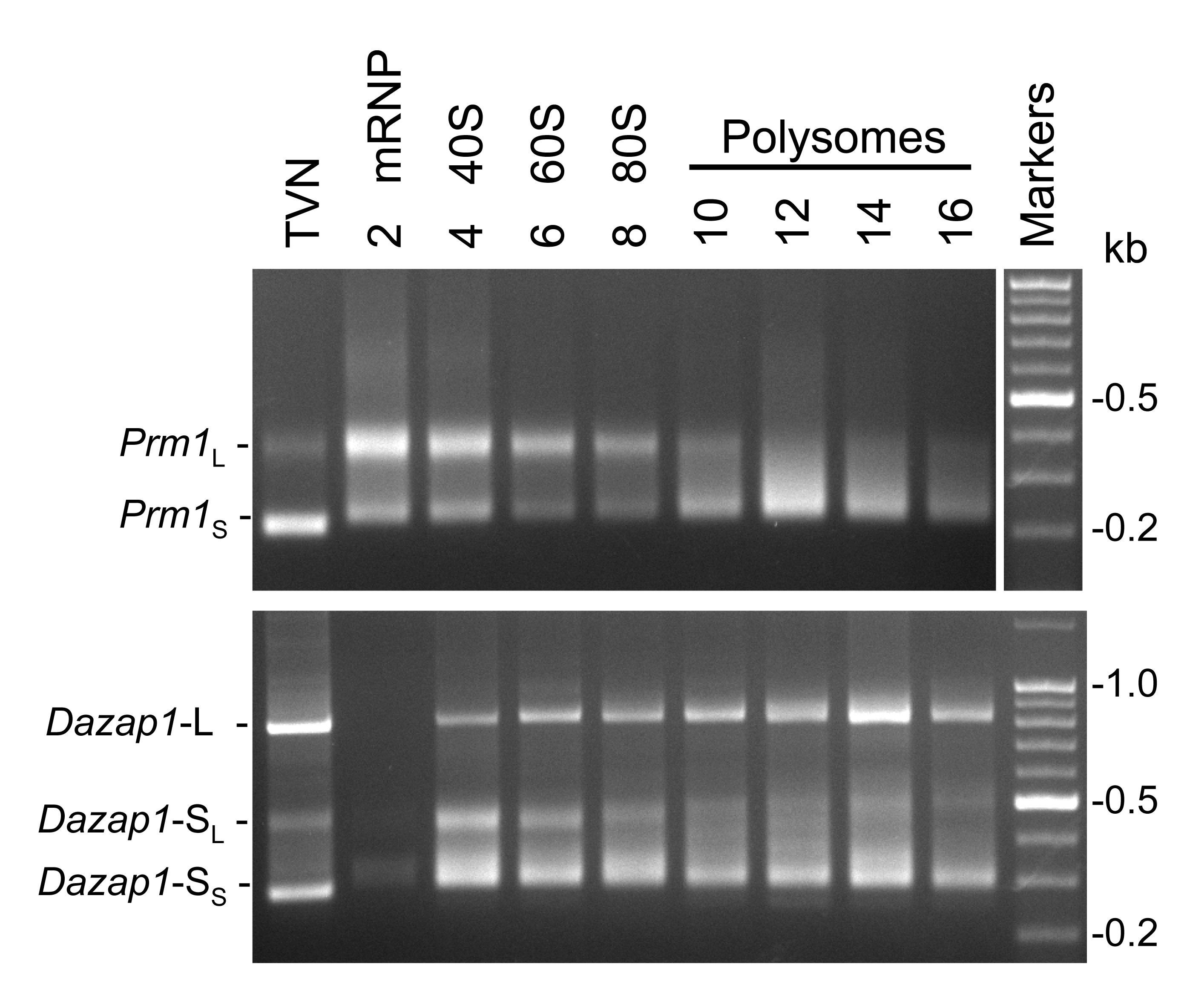


**Figure S1.** **Poly(A) tail length determination of *Prm1* and *Dazap1* transcripts in sucrose gradient fractions.**

Adult mouse testis lysate was fractionated on a 10-50% sucrose gradient and the same proportions of RNAs isolated from alternating fractions were subjected to ePAT and analyzed on a 2% agarose gel. The type of ribosome present in each fraction is indicated at the top. The major bands in the TVN lanes contain an invariant 12-(A) poly(A) tail. Transcripts with long and short poly(A) tails are indicated with L and S subscript, respectively.
