# Supplementary material for: Differential Translation of Dazap1 Transcripts during Spermatogenesis
Source: PLoS One. 2013 Apr 26;8(4):e60873. doi: 10.1371/journal.pone.0060873 (PMC3637229; doi:10.1371/journal.pone.0060873)
Supplement: Figure S2 — DAZL expression level in luciferase reporter assays. The upper panels show western blots of lysates of 3T3 cells cotransfected with pDAZL-Flag and different 3′UTR luciferase reporters. DAZL-Flag was detected with an anti-Flag antibody. The α-tubulin level is used as a loading control. The bottom panel shows quantification of the western blot signals. DAZL-Flag signals were normalized to the tubulin signals. The results represent the averages of three independent experiments. (DOCX) [file pone.0060873.s002.docx]

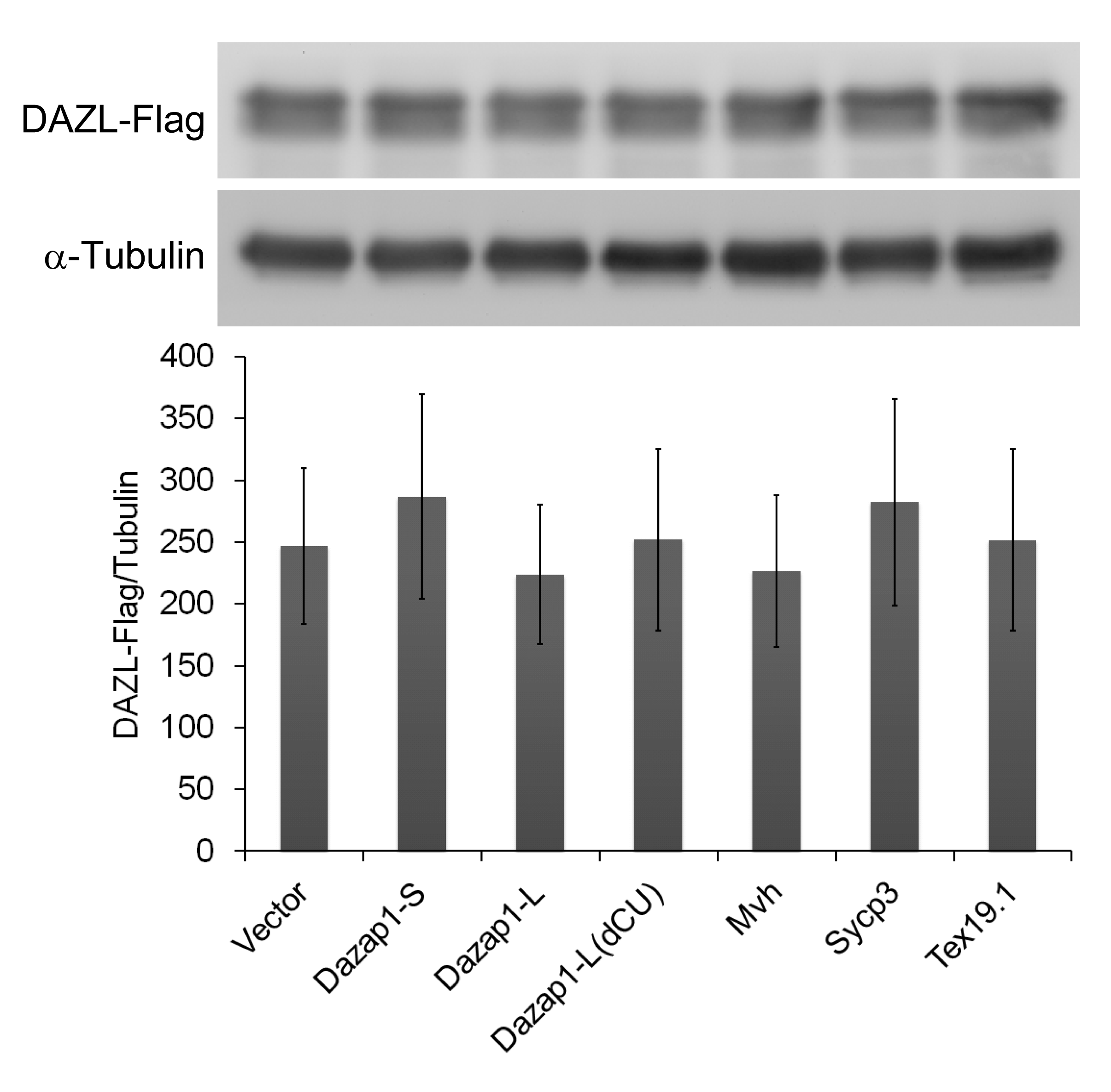


**Figure S2.** **DAZL expression level in luciferase reporter assays.**

The upper panels show western blots of lysates of 3T3 cells cotransfected with pDAZL-Flag and different 3’UTR luciferase reporters. DAZL-Flag was detected with an anti-Flag antibody. The α-tubulin level is used as a loading control. The bottom panel shows quantification of the western blot signals. DAZL-Flag signals were normalized to the tubulin signals. The results represent the averages of three independent experiments.
