# Supplementary material for: Differential Translation of Dazap1 Transcripts during Spermatogenesis
Source: PLoS One. 2013 Apr 26;8(4):e60873. doi: 10.1371/journal.pone.0060873 (PMC3637229; doi:10.1371/journal.pone.0060873)
Supplement: Table S1 — Poly(A) tail lengths of ePAT clones. (DOCX) [file pone.0060873.s003.docx]

**Table S1.** **Poly(A) tail lengths of ePAT clones**

| Transcript | Poly(A) tail length (nt) of individual ePAT clone | | | | | | Average |
| --- | --- | --- | --- | --- | --- | --- | --- |
| *Prm1*_L_ | 113 | 119 | 125 | 113 | 130 |  | 120 |
| *Prm1*_S_ | 22 | 29 | 25 | 24 | 31 | 21 | 25 |
| *Dazap1*-L | 25 | 22 | 32 | 30 | 35 | 23 | 28 |
| *Dazap1*-S_L_ | 116 | 195 | 197 | 146 | 176 | 185 | 169 |
| *Dazap1*-S_S_ | 34 | 24 | 33 | 37 | 34 | 33 | 33 |
| *Sycp3* | 35 | 31 | 27 | 42 | 25 | 36 | 33 |
| *Gapdh*_L_* | 159 |  |  |  |  |  |  |
| *Gapdh*_S_ | 22 | 21 | 20 | 26 | 20 |  | 22 |

* Of 13 clones sequenced, only one clone contained the *Gapdh* sequence.
