# Supplementary material for: Differential Translation of Dazap1 Transcripts during Spermatogenesis
Source: PLoS One. 2013 Apr 26;8(4):e60873. doi: 10.1371/journal.pone.0060873 (PMC3637229; doi:10.1371/journal.pone.0060873)
Supplement: Table S2 — PCR primers for RNAs precipitated from mouse testis lysate with an anti-DAZL antibody. (DOCX) [file pone.0060873.s004.docx]

**Table S2**. **PCR primers for RNAs precipitated from mouse testis lysate with an anti-DAZL antibody.**

| Gene | Primer | Tm (^o^C) | Cycle no. |
| --- | --- | --- | --- |
| *Mvh* | PrMvh-1: 5'-tctagagcaaacgaacatacttcaagtctg | 59 | 28 |
|  | PrMvh-2: 5'-tctagagggaaatgtgtttcatcttttatttgcaag |  |  |
| *Sycp3* | PrSycp3-1: 5'-tctagatgagtctttgaagaaagaacttgaacc | 59 | 28 |
|  | PrSycp3-2: 5'-tctagatttaaatcatctttattgacacaatcgtgg |  |  |
| *Tex19.1* | PrTex19.1-1: 5'-tctagatgcacattcctgagacactaaagc | 59 | 34 |
|  | PrTex19.1-2: 5'-tctagaacaccaactttattcaacaaaagccC |  |  |
| *Prm1** | PrPrm1-1: 5'-accatggccagataccgatg | 54 | 20 |
|  | PrPrm1-2: 5'-gtggcgagatgctcttgaag |  |  |
| *Gapdh** | PrGapdh-1: 5'-atgtgtccgtcgtggatctg | 54 | 28 |
|  | PrGapdh-2: 5'-cctcagtgtagcccaagatg |  |  |

PCR condition: 95 ^o^C 15 min for Taq polymerase activation and then cycling by 94 ^o^C 30 sec, Tm ^o^C 90sec, 72 ^o^C 30-50sec using QIAGEN Multiplex PCR reagent (QIAGEN)

*PCR condition: cycling by 94 ^o^C 30 sec, Tm ^o^C 30 sec, 72 ^o^C 30sec using Taq polymerase (Bioman scientific, Taipei, Taiwan)
