# Supplementary material for: Differential Translation of Dazap1 Transcripts during Spermatogenesis
Source: PLoS One. 2013 Apr 26;8(4):e60873. doi: 10.1371/journal.pone.0060873 (PMC3637229; doi:10.1371/journal.pone.0060873)
Supplement: Table S3 — Gene-specific ePAT forward primers. (DOCX) [file pone.0060873.s005.docx]

**Table S3**. **Gene-specific ePAT forward primers**

| Gene | Primer | Tm (^o^C) | Cycle no. |
| --- | --- | --- | --- |
| *Prm1* | PrPrm1-PAT: 5'-cataaggtgtaaaaaatactagatgcacagaatag | 59 | 24 |
| *Dazap1* | PrDAP176:5’-agtggcttcggacgcgggcagaaccacaac | 64 | 32 |
| *Dazap1*-L | PrDAP1-31: 5'-ctcgagattattttcttgagcc | 57 | 30 |
| *Sycp3* | PrSycp3-1: 5'-tctagatgagtctttgaagaaagaacttgaacc | 59 | 28 |
| *Gapdh* | PrGapdh-1: 5'-atgtgtccgtcgtggatctg | 57 | 28 |

PCR condition: 95 ^o^C 15 min for Taq polymerase activation and then cycling by 94 ^o^C 30 sec, Tm ^o^C 90sec, 72 ^o^C 30-50sec using QIAGEN Multiplex PCR reagent (QIAGEN)
